# Research on Vibroactivity of Toothed Gears with Highly Flexible Metal Clutch under Variable Load Conditions

**DOI:** 10.3390/s23010287

**Published:** 2022-12-27

**Authors:** Mariusz Kuczaj, Andrzej N. Wieczorek, Łukasz Konieczny, Rafał Burdzik, Grzegorz Wojnar, Krzysztof Filipowicz, Grzegorz Głuszek

**Affiliations:** 1 Department of Mining Mechanisation and Robotisation, Faculty of Mining, Safety Engineering and Industrial Automation, Silesian University of Technology, 44-100 Gliwice, Poland; 2 Department of Road Transport, Faculty of Transport and Aviation Engineering, Silesian University of Technology, 40-019 Katowice, Poland

**Keywords:** variable load, sensors, vibration, gears, flexible torsion clutch

## Abstract

The article provides a discussion on a methodology intended for testing of power transmission systems featuring an innovative highly torsionally flexible metal clutch patented by the co-authors of this paper. What this methodology takes into account is the amplitude and frequency analyses discussed in the article, as well as a sensing system based on diverse piezoelectric and magnetic phenomena, the Doppler effect, etc. Both contact and non-contact (laser measurement) methods were used during the tests. The purpose of the tests conducted at the stand, originally designed by the authors in accordance with the methodology proposed, was to evidence that using the innovative and patented, highly torsionally flexible metal clutch makes it possible to reduce the vibrations of multi-stage toothed gears, consequently reducing the forces affecting the gear bearings and those acting at the tooth space, which is to enable the service life of individual components of the power transmission systems intended for mining scraper conveyors to be significantly extended. Based on the studies and analyses performed by the authors, one can observe and conclude that the methodology proposed in the paper makes it possible to use an example of a relatively complex power transmission system in order to examine the relationships between the processes at work, i.e., the decline of the linear vibrations of the gear housing (which is undoubtedly positive in power transmission systems) at the expense of increasing torsional vibrations of the innovative clutch, the latter not to be considered unfavourable to users in the case analysed.

## 1. Introduction

In order to ensure adequate performance and, by that means, also cost-effectiveness of businesses, machines and their subassemblies are expected to be robust and durable in operation [1,2,3,4]. However, this is particularly difficult to achieve when machinery is exposed to aggressive environmental factors. Such factors [5] may include abrasive agents, chemical compounds, and variable instantaneous loads which can overload machine components, thus accelerating their degradation. These detreminants of process systems can be involved in synergistic interactions [6,7], and their outcome can be significantly greater. Insofar as the former two factors are typically counteracted by technological means or by way of material conversion [8,9,10,11,12,13,14], the latter component is very difficult to eliminate. This observation applies particularly to the components of the power transmission systems used in process machinery, such as toothed gears. Where this is the case, work may proceed in unsteady states resulting from the occurrence of instantaneous overloads and start-ups. The highlights of this study include ensuring adequate operating conditions for the gears used in the power transmission systems of scraper conveyors. At this point, it should be pointed out that the aforementioned conveyors are among the most critical pieces of machinery in the process systems of mining plants as well as in the coal handling systems of power plants, and furthermore, they often operate under unsteady conditions, comprising start-ups, overloads, chain slackening, etc.

The factors which affect the vibroacoustic condition of toothed gears may be attributable to both external and internal causes. One can distinguish between three groups of internal factors affecting the vibroacoustic condition of gears [15,16,17,18].

- Structural factors, including nominal rotational speed, nominal gear mesh frequency, nominal gear load, damping properties of lubricants, fit and stiffness of bearings, structural form of the gear housing, and free vibrations of the entire system; for this group of factors, it is possible to make sure that various causes of damage and malfunction can be significantly reduced at the design stage, for instance by increasing dimensions or reducing breaking stresses.
- Technological factors, including pitch deviations, tooth line direction deviations, radial runout, and surface irregularities; a common solution to this group of problems, one which reduces the potential damage, is increasing the gear teeth manufacture accuracy, for instance by using advanced machining equipment or highly accurate assembly.
- Disruptive factors interfering with the motion of gears, including change of meshing stiffness and damping in the mesh within the engagement section, tooth deformation due to loading, dynamic imbalance of wheels and shafts, change in wheel interaction conditions attributable to wear of tooth surfaces, inter-tooth play; it is relatively difficult to curb the negative effects of the aforementioned constituent factors, and it often requires reference profile alterations, adequate outline modifications or even gear overhaul.

One must consider certain external forces at play, mainly including start-ups, load changes, unbalance, and assembly errors across the entire power transmission system. It is possible to counteract the adverse effects of the said dynamic excitations of the power transmission system components through continuous vibroacoustic monitoring [19,20,21,22,23,24,25,26,27,28,29,30,31,32,33,34,35,36,37,38] oriented towards the elimination of the machine’s operating states which cause instantaneous overloads of its components, as well as by using clutches enabling the system’s pulse excitations to be reduced. With regard to the clutches themselves, their reduction capabilities are closely linked with the more flexible mechanism applied [39,40,41,42,43,44,45,46,47,48,49]. On the other hand, the vibroacoustic monitoring system must record changes in the parameters of multiple processes and should not rely solely on contact-mounted vibration acceleration sensors, and the latter problem has been discussed in more detail further on in this paper.

**Figure 1:**
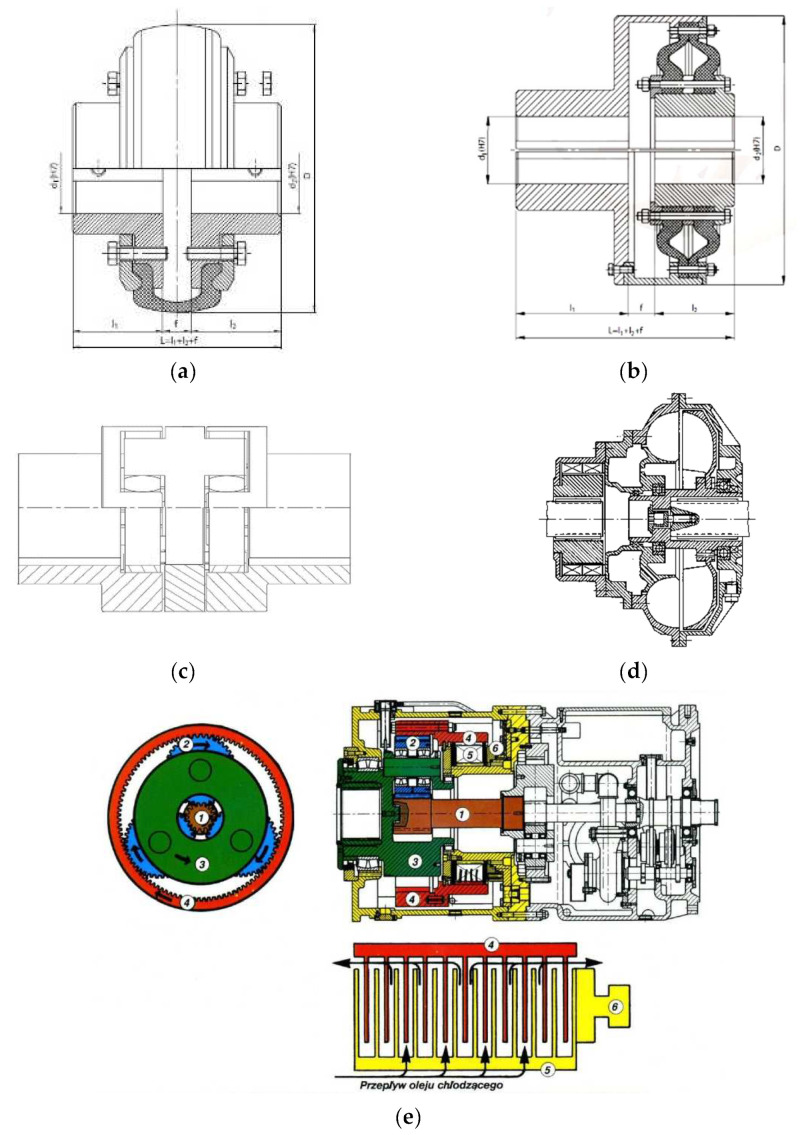
Schematic diagrams of different clutch types: (**a**)—tyre clutch, (**b**)—diaphragm clutch, (**c**)—double-insert clutch, (**d**)—hydrokinetic clutch, (**e**)—CST multi-disc clutch integrated with a planetary gear; designations: 1—central gear wheel, 2—planet wheels, 3—cage with output shaft, 4—internal gear wheel with moving coupling disks, 5—stationary coupling discs, 6—actuator [50].

Rigid and toothed clutches are characterised by very low flexibility, which does not allow reduction in the dynamic forces occurring in power transmission systems. Tyre clutches (Figure 1a) or diaphragm clutches (Figure 1b) make use of the significant deformability of rubber under the load torque which affects gear shafts, but in their case, the load transfer capabilities are very limited.

Insert clutches (Figure 1c) are very often used in the drive systems of scraper conveyors. They are capable of transferring a maximum torque of up to 15,000 Nm. The shape of both the hub claws and the flexible insert ensures optimum interplay under conditions of misalignment between motor shafts and the device being driven. These clutches are also characterised by certain torsional flexibility, but in most cases it is insufficient to minimise the varying loads caused by either start-up or overload.

Hydrokinetic clutches are considered to be the power transmission components which enable soft starting of scraper conveyors. The foregoing was confirmed in a study by Dolipski et al. [51,52,53,54]. The transfer of torque from the active member, referred to as a pump rotor, to the passive member, i.e., a turbine rotor (Figure 1d), is performed hydraulically in hydrokinetic clutches by making use of the change in the kinetic energy of the fluid circulating between the two rotors [55]. The coupling elements used most commonly are constant-fill couplings, with or without a retarding chamber, as well as flow couplings. According to Suchoń [56], a disadvantage of these couplings is inadequate protection of power transmission system components against sudden locking (stall), which pertains to the gear in particular. On account of the long time of transition to the steady state of operation (even up to 30 s), the couplings of this type are not suitable for the drive systems which require frequent start-ups and which do not reduce the dynamic loads emerging in operation.

An example of the use of a friction clutch in a power transmission system (Figure 1e) is the controlled start transmission (CST) system, which integrates two power transmission components, i.e., the gear and the multi-disc friction clutch. This system ensures load-free start-up of all drive motors, and it balances the load in the system in the course of steady-state operation. On the other hand, in the event that the drive is locked (overloaded), the device’s control unit commands the relevant actuators to completely disconnect all drives. However, the CST system’s design is very complex, which also significantly increases the probability of the power transmission system failure. The study of friction clutch-based drive systems by Skoć and Drwięga [57] revealed the importance of the heat capacity of the clutch discs for transfering the loads occurring in the course of frequent start-ups, but also the dependence of clutch shaft’s resistance in the uncoupled state on residual friction, and the fluctuation of disc friction coefficient in the coupling process.

**Figure 2:**
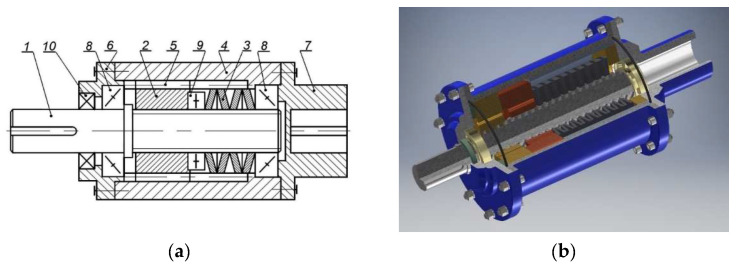
Design (**a**) and example (**b**) of a torsionally flexible metal clutch [62]; designations: 1—clutch shaft, 2—sliding sleeve, 3—set of disc springs, 4—clutch housing, 5—moving splined coupling, 6—cover, 7—clutch hub, 8—cone bearings, 9—thrust bearing, 10—sealing ring.

A completely new approach to the attenuation of start-ups, dynamic changes and overloads in scraper conveyor systems was presented by Kowal and Filipowicz [58,59,60]. These authors proposed to use a screw system aided by a set of springs to form a flexible element characterised by a very high relative torsional angle of both coupling members (Figure 2). Such a design of flexible couplings has never been used in the operating practice of process machines to date. In his own paper, Filipowicz [61] discusses the possibility of two-directional operation of flexible metal clutches.

The operating principle of a torsionally flexible metal clutch is that the running torque acts on the active side of the clutch directly via its shaft (1) and is then transmitted to a sliding sleeve (2) by means of a multiple thread mechanism. The increasing torque causes the shaft (1) to rotate against the sleeve (2) and, at the same time, against the clutch housing (4). The axial force generated in the thread mechanism initiates the sleeve’s sliding movement along the shaft axis (clutch axis). The sleeve’s movement is restricted to plane motion by the moving splined coupling (5) between the sleeve (2) and the clutch housing (4). At the same time, the sleeve’s plane motion causes compression of the set of disc springs (3) adequately chosen to match the assumed clutch characteristics. The compression of the springs generates an internal elastic strain force affecting this spring set. At each momentary fixed position of the sliding sleeve, this force balances the axial force generated in the thread mechanism, which results from an external running torque. Thus generated, the balance of forces in the clutch’s thread mechanism, defined by the momentary fixed position of the sliding sleeve (2) against the shaft (1) and housing (4), is also defined by the angle of relative rotation of the clutch members—the active and the passive one, at which the instantaneous value of the running torque is “transferred” from the active to the passive side of the clutch. Any instantaneous overloading of the power transmission system by the running torque causes additional compression of the clutch’s elastic components, while load reduction releases the compressive force. Once the power transmission system has been fully unloaded, the sliding sleeve (2), compressed by the gradually relaxing spring set, returns to its initial design position relative to the clutch shaft axis.

Filipowicz has demonstrated [62,63,64] that the flexible metal clutch can effectively attenuate the torque impulses generated by the braking system. A report by Wieczorek et al. [65] confirmed the high durability of the screw-nut system against friction. Furthermore, Wojnar et al. [66] proposed a method for advanced time–frequency analysis and for order analysis of vibrations measured at the support bearing housing for a flexible and locked clutch.

The problem of transverse and torsional vibrations is very topical and widely addressed in the literature. For instance, in paper [67], the properties of the eigenvalues and eigenmodes for transverse and torsional vibrations of a mechanical system where two of the three component bars are identical have been defined. The determination of these properties allows the calculus effort and the computation time and thus increases the accuracy of the results in such matters. For such kind of structure, we have demonstrated properties of the eigenvalues and eigenvectors that allow ease and simplify the calculation of real structures. Paper [68], on the other hand, points out that in many technical applications (such as those in automotive engineering or in structural mechanics), the mechanical system studied can be considered as one which is composed of two or many identical subsystems or parts. These kinds of symmetries of the structure can be used in order to simplify the analysis of the vibrations and make it possible to reduce the dimension of the differential equations that describe the motion. Paper [69] addresses one rotational and two translational degrees of freedom for the rotor and a single arclength degree of freedom for each absorber, considered in the planar model. The well-defined structure of the vibration modes is obtained by analytical and numerical investigations of the associated eigenvalue problem. This vibration mode structure is similar to that for CPVA systems with equally spaced, identical absorbers. Thus, the disrupted symmetry from multiple absorber groups does not destroy the vibration mode structure resulting from the cyclic symmetry within each group. The critical speeds and flutter instability of the system are investigated. The computational approach is particularly valuable, however, on account of the multitude of unknown vibration damping and rigidity coefficients; the experimental approach was chosen for purposes of this study.

However, the foregoing studies do not address the vibroactivity of toothed gears used in scraper conveyor power transmission systems coupled with a flexible metal clutch of a novel design. Additionally, the authors identified the need for the detection of torsional vibrations of the spring clutch itself. The foregoing observations provided grounds for the objectives of this study, namely to develop a comprehensive method for measuring vibrations, including torsional vibrations of highly flexible metal clutches and linear vibrations of gear housings, as well as for measuring other physical parameters and recording useful signals, which is a method that enables detailed analysis of a new and patented highly flexible metal clutch and makes it possible to prove that using this clutch in a power transmission system featuring a toothed gear can successfully reduce its vibrations.

The novel aspect of the study is also that the authors made an attempt to illustrate the relationships between the oscillatory processes observed in the subject of the tests as well as to demonstrate the effective operation of the clutch patented by two of the co-authors. Furthermore, our intent was to discuss the methodology of testing of such innovative power transmission systems and to show that using highly diversified measuring methods and sensors is necessary.

Both the goal and the scope of the research, as defined above, clearly imply the innovative nature of the study, since the properties of toothed gears coupled with highly flexible metal clutches, featuring a screw-and-nut type torsional system and a spring-type tensioning system, have not been studied under near real-life conditions to date.

## 2. Materials and Methods

**Figure 3:**
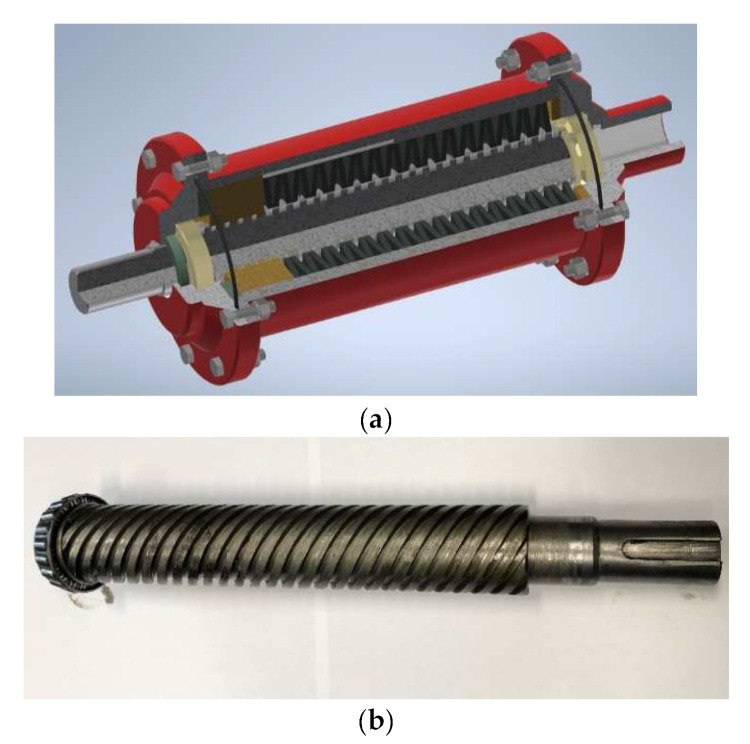
Design of the flexible metal clutch: (**a**) model, (**b**) actual screw.

The flexible metal clutch (Figure 3) was characterised [65] by the following parameters:

torsional stiffness—135 Nm/deg,
dimensionless damping coefficient ψ—0.52,
nominal torque transferred—600 Nm,
safety factor—3.

**Figure 4:**
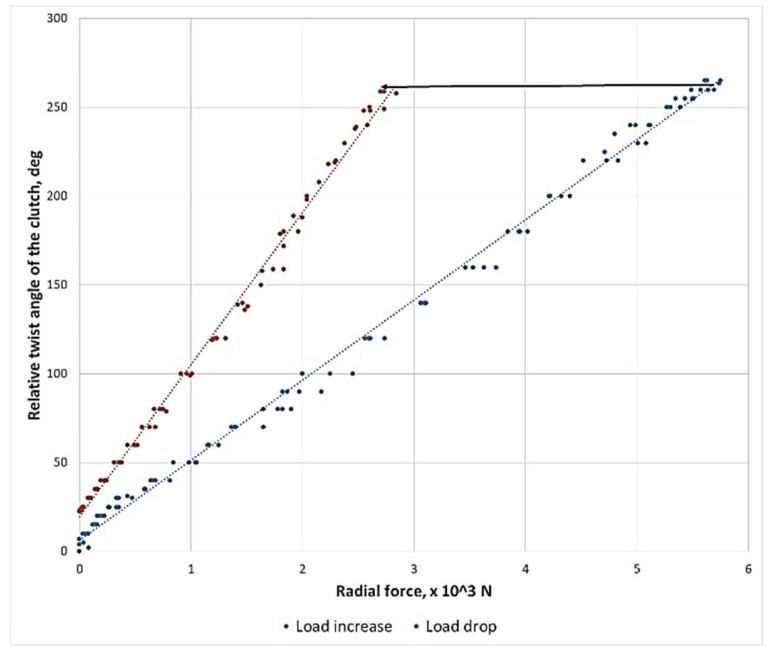
Static characteristics of the flexible metal clutch subject to testing.

The clutch in question operated in two modes: flexible and non-flexible. The static characteristics of the clutch for the flexible operating mode have been provided in Figure 4. It is rather evident that, under static conditions, the clutch is characterised by a certain hysteresis caused by the friction between the screw and the nut, causing a phase of higher clutch flexibilisation relative to the current load to emerge while load is being relieved.

**Figure 5:**
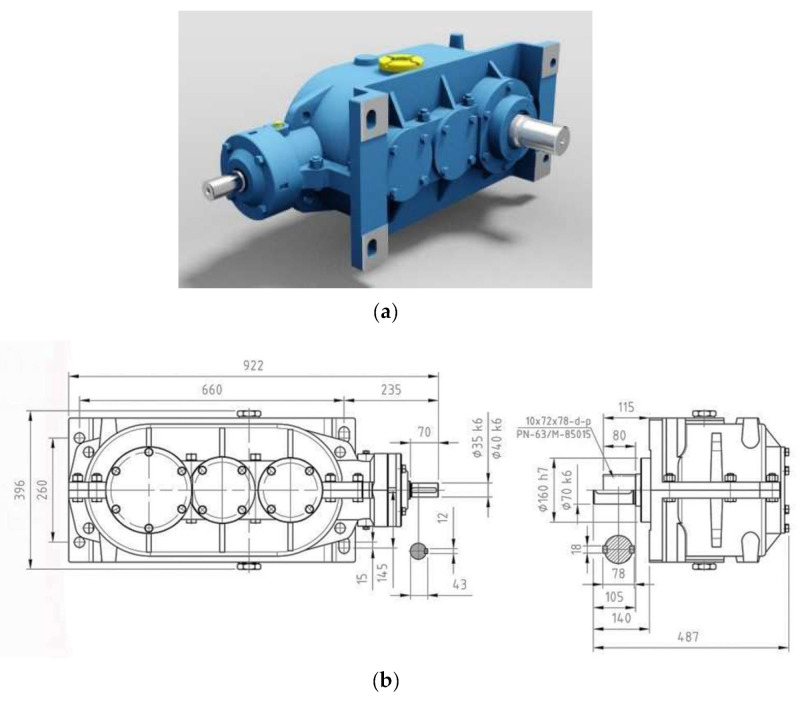
Type PAT-KW3 toothed gear: (**a**) schematic diagram, (**b**) connection dimensions—front and right side view [70].

The impact of a highly torsionally flexible metal clutch on the level of housing vibrations under conditions of time-varying dynamic loads was examined using a toothed gear typical of scraper conveyors (Figure 5), having the following characteristics [70]:

long-term power transmitted (at 1470 rpm)—22 kW,
three-stage gear (1 conical and 2 cylindrical stages),
total gear ratio i = 12.962,
splash lubrication with a VG 220 grade mineral oil.

**Figure 6:**
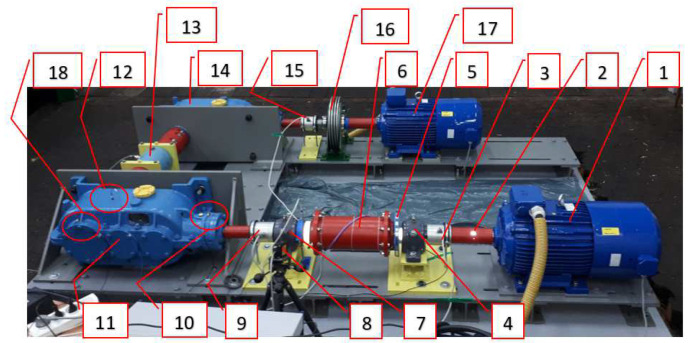
Test stand; designations: 1—driving motor, 2—stiff sleeve clutch, 3, 9, 13, 15—torque meters (respectively: M1, M2, M4, M3), 4, 7—bearing support, 5—relative rearrangement angle sensor for active and passive coupling members, 6—torsionally flexible metal clutch, 8—Polytec RLV-5500 laser vibrometer, 10—gear’s input shaft bearing housing, 11—multistage reduction gear (subject to testing), 12—uniaxial vibration acceleration transducer, 18—triaxial vibration acceleration transducer, 14—multistage mulitplying gear, 16—powder brake, 17—braking motor.

The proper studies of the gears in question were preceded by determining the effect of the rotation direction on the housing vibrations generated. While conducting tests, no measurable differences were detected either for the vibration acceleration values previously obtained or for the frequency spectrum. This was due to the fact that the gear in use was characterised by a very low ratio of the bevel stage, which caused no significant effect on the responses observed in the bearings. Tests were conducted at a test stand (Figure 6) enabling variable loads to be generated.

The gears subject to testing were mounted at the test stand in an identical manner to that in which they are arranged in in a lightweight scraper conveyor. This made it possible to run the vibroactivity measurements on their housings under near real-life conditions.

The relevant load variables were generated by way of instantaneous changes in frequency applied at the braking motor inverter, whose behaviour corresponded to a rectangular function (No. 17, Figure 6). This triggered a change in the motor’s rotational speed and, by that means, also a unilaterally pulsating load on the power transmission system subject to testing. As mentioned above, a rectangular input function was applied during the tests, causing changes in the torsional angle of the clutch members in the power transmission system examined, as discussed in detail in Section 3 of this article. The mean values of the load torque and of the amplitude of the load torque changes thus induced in the power transmission system in question between the flexible clutch and the multi-stage reduction gear as well as the operating modes of the clutch have all been provided in Table 1. The period of the load torque changes was set at 2 s (50% for low state and 50% for high state) on the basis of the article authors’ long-term experience in the specifics of the operation of mining conveyors.

On account of the need to reduce the impact of potential inverter interference on the measurements, dedicated anti-aliasing filters were designed and implemented.

**Figure 7:**
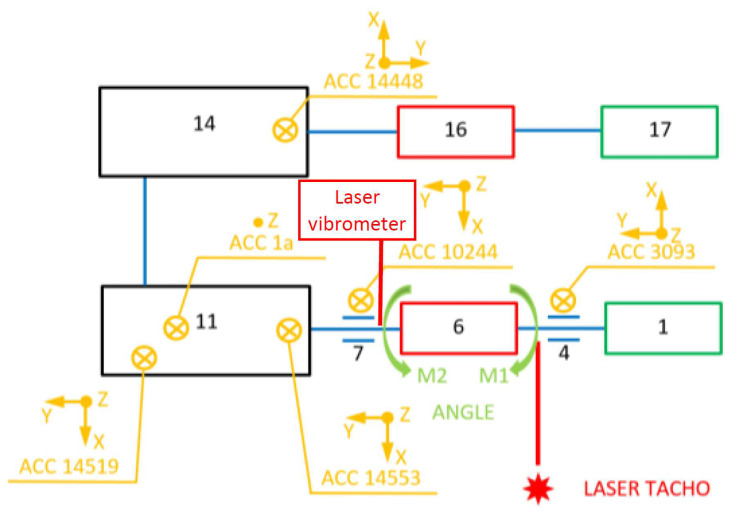
Arrangement of measurement points; designations: 1—driving motor, 4, 7—bearing support, 6—torsionally flexible metal clutch, 11—multistage reduction gear (subject to testing), 14—multistage mulitplying gear, 16—powder brake, 17—braking motor.

For purposes of the identification studies of the flexible metal clutch, a new method was developed based on the following:

- Torque measurement using torque meters M1, M2, M4 (No. 3, 9, 15—Figure 6) (MT-750, Pracownia Elektroniki R. Pomianowski, Poznań, Poland) set up- and downstream the flexible clutch as well as at the output of the gear performing the multiplier function; the measurement signal from the torque meter sensor was conditioned in a matching circuit, transmitted to a three-channel measuring system, and recorded in parallel with the other signals recorded;
- Torsional angle measurement—this measurement was taken using a magnetic sensor which generated a signal related to the rotation of a measuring disc featuring a wound bipolar magnetic tape (add designation, Pracownia Elektroniki R. Pomianowski, Poznań, Poland); the measurement signal received from this sensor was conditioned in the matching circuit, transmitted to a single-channel measuring system, and recorded in parallel with the other signals recorded;
- Measurement of vibration accelerations on the gear housing by contact methods using four Endevco 44A10 3-axis accelerometer sensors (measuring points: 14519, 14553, 10244, 14448—Figure 7) and one Dytran 3093 3-axis accelerometer sensor;
- Non-contact laser measurement of the instantaneous changes in the torsional vibration velocity of the flexible clutch’s input shaft using the Polytec RLV-5500 Rotational Laser Vibrometer (Waldbronn, Germany);
- Determination of the impulses associated with the shaft rotation using two ROLS (tacho) laser sensors with a power of <1 mW and a measuring frequency of 1–250,000 rpm.

Three Sirius cards (from Dewesoft, Trbovlje, Slovenia) were used for recording and analysis purposes with 24-bit 16-channel synchronous sampling using DEWESOFT software (from Dewesoft, Trbovlje, Slovenia). A sampling frequency of 20 kHz was applied during the measurements. The arrangement of the measuring sensors at the test stand has been shown in Figure 7. 

As mentioned above, in order to provide the most comprehensive presentation of the relationships between the oscillatory processes observed in the subject of the tests as well as to demonstrate the effective operation of the clutch patented by two of the authors of this article, next to the conventional relays of vibration accelerations as well as the torque meters and the magnetic sensor used for angle measurement, as described in the previous section, also a concept of contactless laser measurement of the velocity of torsional vibrations, significantly less popular in measurements, was used under the studies.

**Figure 8:**
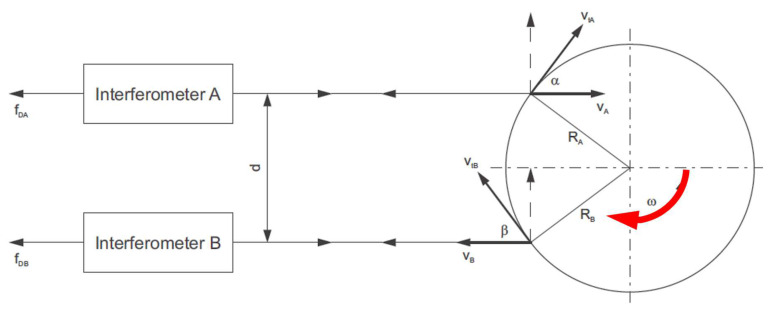
Schematic diagram illustrating the operating principle of a laser angular velocity relay.

**Figure 9:**
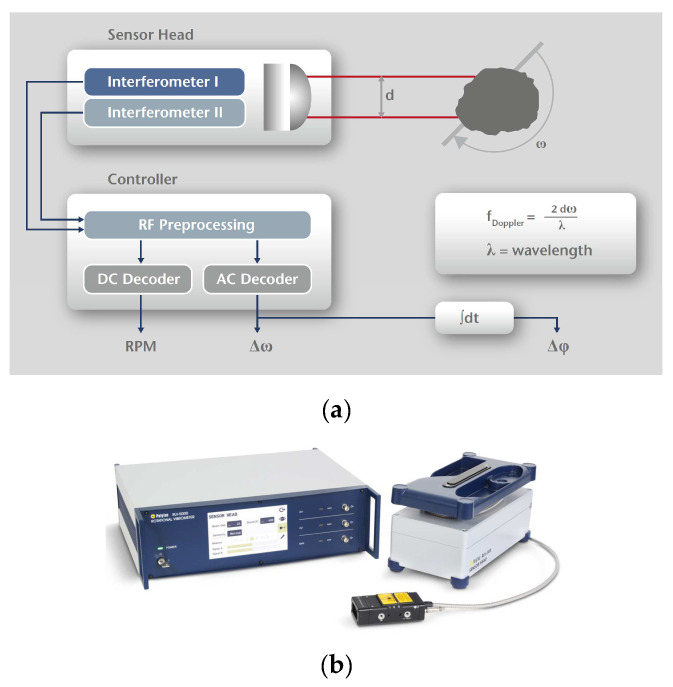
Visual representation of the processing of signals (**a**) using the Polytec RLV-5500 rotational laser vibrometer (**b**)—see Figure 6, item No. 8 [72,73].

Figure 8 (drawn up with reference to [71]) visually represents the operating principle of a rotational laser vibrometer based on the Doppler effect, similarly to the laser vibrometers used to measure linear vibrations (one- and three-dimensional), the difference being that the rotational laser vibrometer uses two parallel laser beams enabling measurements of velocities *V_A_* and *V_B_*. Where this is the case, the following relationships hold:

(1) $$V_{A}=V_{tA}\cdot \cos\alpha_{La}=\omega \cdot R_{A}\cdot \cos\alpha_{La},$$

(2) $$V_{B}=V_{tB}\cdot \cos\beta_{La}=\omega \cdot R_{B}\cdot \cos\beta_{La},$$

where *ω* angular velocity; designations of the remaining variables have been provided in Figure 8. Based on these velocities and in accordance with relationship (3), one can determine angular velocity *ω* of the shaft, and on such grounds, also instantaneous angular velocity changes Δ*ω* (Figure 9).

(3) $$\omega =\frac{V_{A+}V_{B}}{d},$$

where *d* is fixed separation of laser beams resulting from the measuring head’s design.

The most important parameters of the rotational laser vibrometer used in the studies addressed in this paper have been summarised in Table 2.

Operating temperature: +5 °C–+40 °C. Laser type: Helium-Neon, 633 nm (red). Laser output: <1 mW per beam, class 2. Other detailed parameters of the RLV-5500 laser angular velocity relay (laser vibrometer) can be found in papers [68,70]. The rotational laser vibrometer used under the studies in question was characterised by the separation of beams oriented towards the object of *d* = 7.5 mm and by the sensor head’s distance to the measurement surface of 400 mm.

## 3. Results and Discussion

**Figure 10:**
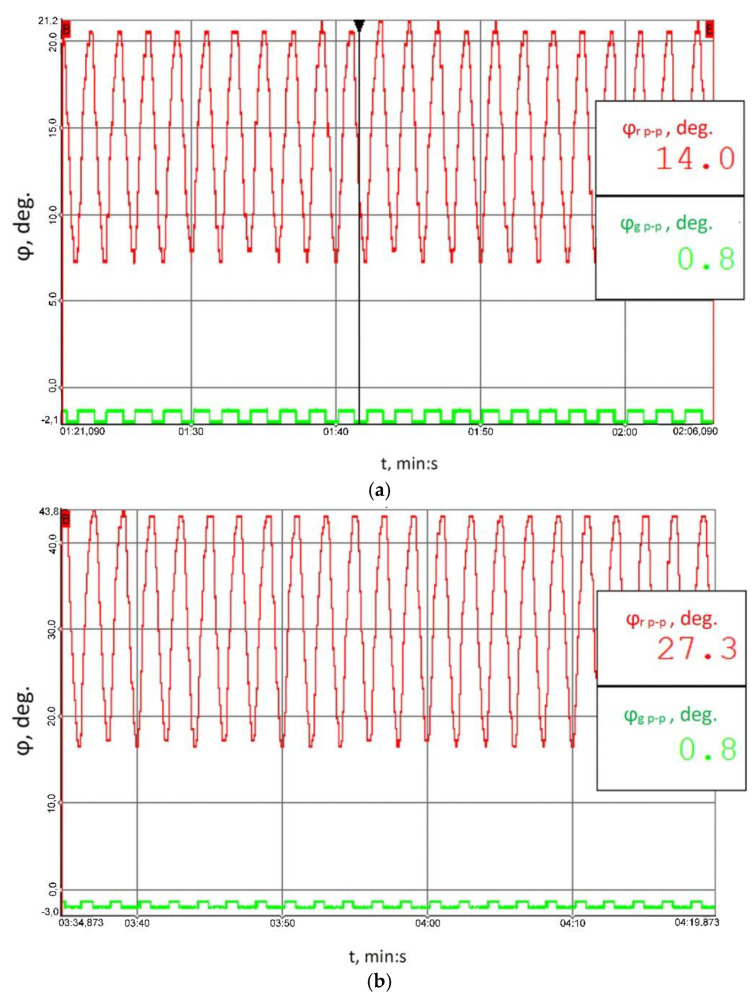

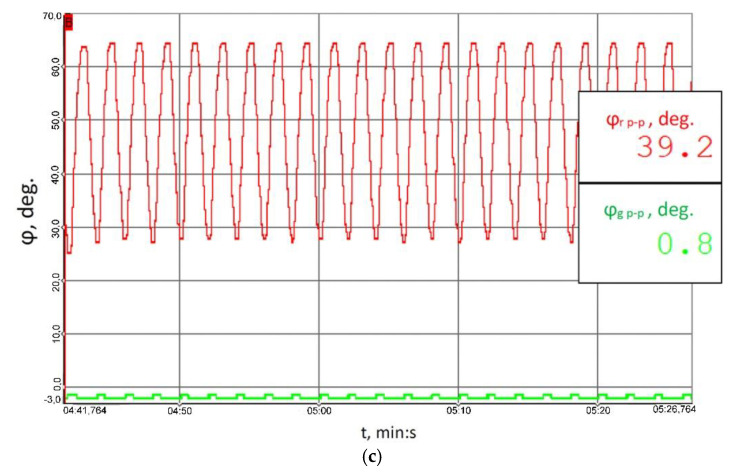
Recorded time histories of the torsional angle of the clutch members for the following load scenarios: (**a**) 41.8 ± 27.9 Nm, (**b**) 79.2 ± 55 Nm, (**c**) 114.8 ± 75.9 Nm; red colour marks the results obtained for the flexible operation mode of the clutch; green colour corresponds to the locked operation mode.

Figure 10 illustrates the recorded behaviour of the relative torsional angle of the clutch members (ϕ) in the function of time. Under the impact of the applied time-varying load (Table 1), with respect to the flexible clutch operation (red colour), the peak-to-peak values of the changes in the relative torsional angle of the clutch members came to 14.0°, 27.3°, and 39.2° (the uncertainty of the angle determination did not exceed ± 1.7° for all the cases analysed). These values corresponded to the load values of 41.8 ± 27.9, 79.2 ± 55, and 114.8 ± 75.9 Nm, respectively (the uncertainty of torque determination did not exceed ± 1.2 Nm for all the cases analysed). For the clutch in the locked condition, constant small angle changes with the peak-to-peak value of 0.8° could be observed, corresponding to the circumferential backlash of individual power transmission system components, and of multi-stage gears in particular. Given that the amplitude in the changes is particularly relevant to the dynamic operation, before the measurements of the torsional angle of the locked clutch members started, it was not necessary to reset the system used to record the torsional angle of the clutch members, and hence the negative values which Figure 10 shows for the case of the clutch’s locked operation mode (green colour).

**Figure 11:**
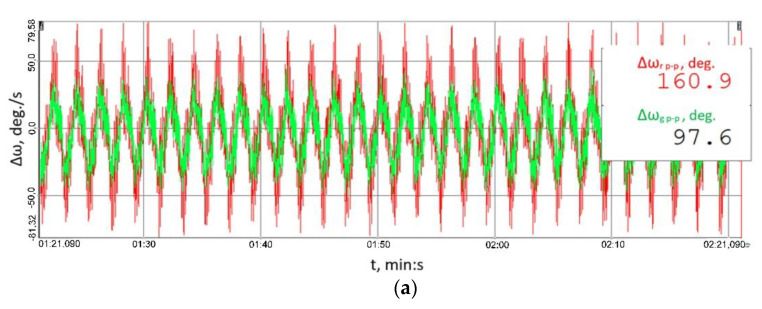

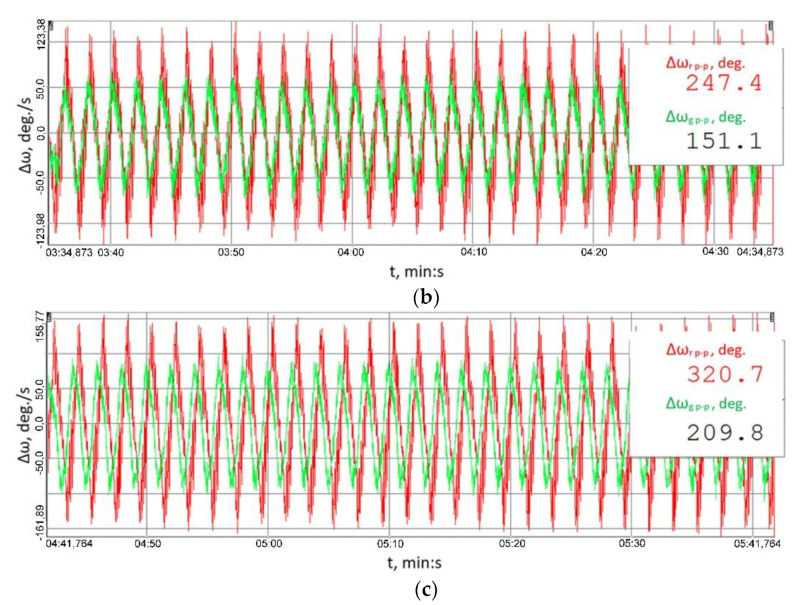
Time histories of instantaneous changes in the velocity of the torsional vibrations of the shaft between the clutch and the multi-stage reduction gear recorded by a contactless method for the following load scenarios: (**a**) 41.8 ± 27.9 Nm, (**b**) 79.2 ± 55 Nm, (**c**) 114.8 ± 75.9 Nm; red colour marks the results obtained for the flexible operation mode of the clutch; green colour corresponds to the locked operation mode.

The changes in the torsional angle of the clutch members shown in Figure 10 generate instantaneous changes in the angular velocity of the shaft (Figure 11) situated between the multi-stage reduction gear (No. 11 in Figure 6) and the clutch, since it is this multi-stage reduction gear that transfers the time-varying load torque generated by the braking motor (No. 17 in Figure 6). The higher peak-to-peak values of the instantaneous changes in the velocity of the torsional vibrations of the flexible clutch’s input shaft recorded by a contactless method using a rotational laser vibrometer (RLV-550, Polytec, Waldbron, Germany) for each of the loads tested correspond to the flexible operation mode of the clutch. The foregoing is attributable to the fact that, at times when the load increases, it is the flexible clutch members that are subject to torsion, and this causes larger instantaneous changes in the angular velocity of the shaft analysed.

**Figure 12:**
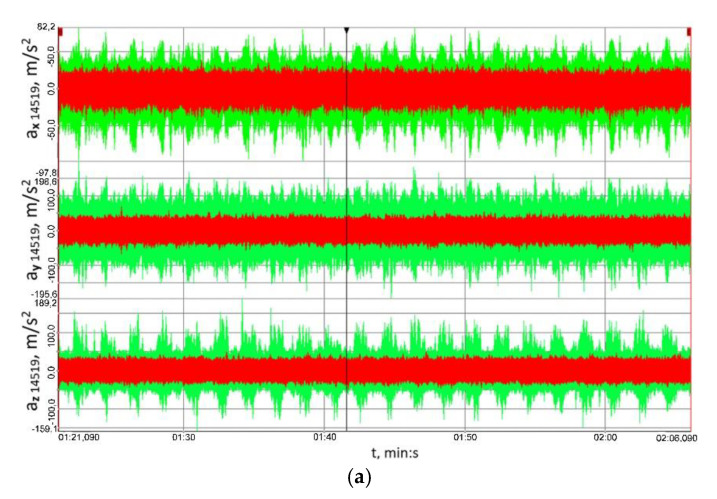

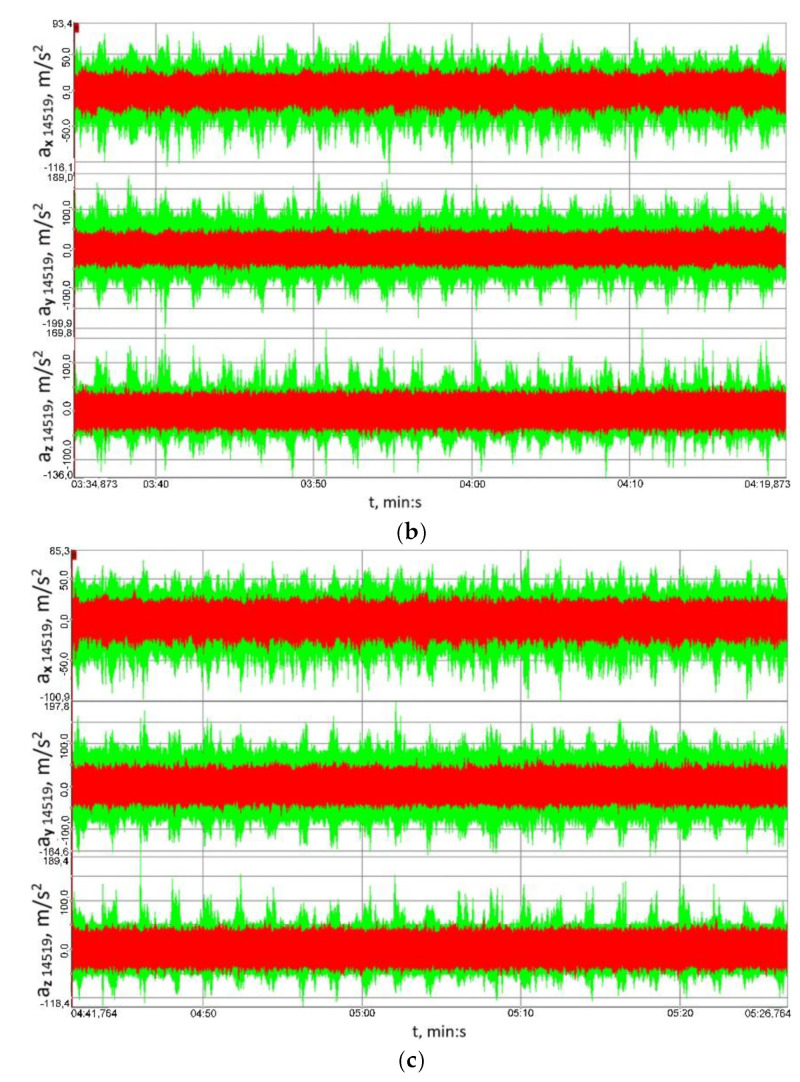
Time histories of the vibration accelerations recorded in three mutually perpendicular measurement directions using transducer No. 14519 (Figure 7) attached to the housing of the output shaft bearing in the multi-stage reduction gear: (**a**) 41.8 ± 27.9 Nm, (**b**) 79.2 ± 55 Nm, (**c**) 114.8 ± 75.9 Nm; red colour marks the results obtained for the flexible operation mode of the clutch; green colour corresponds to the locked operation mode.

**Figure 13:**
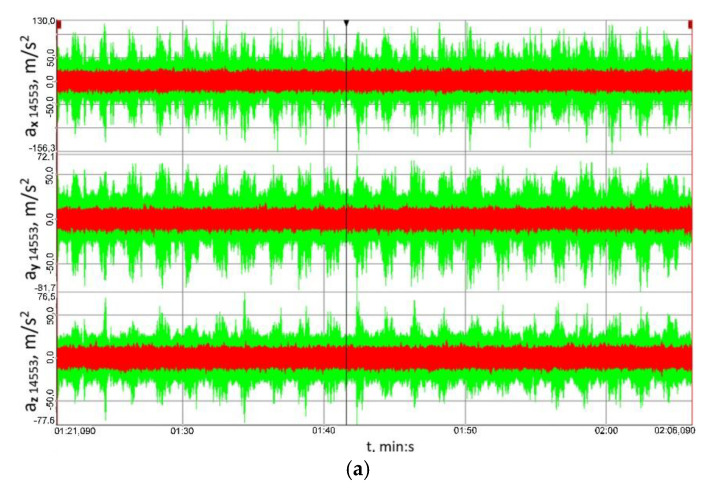

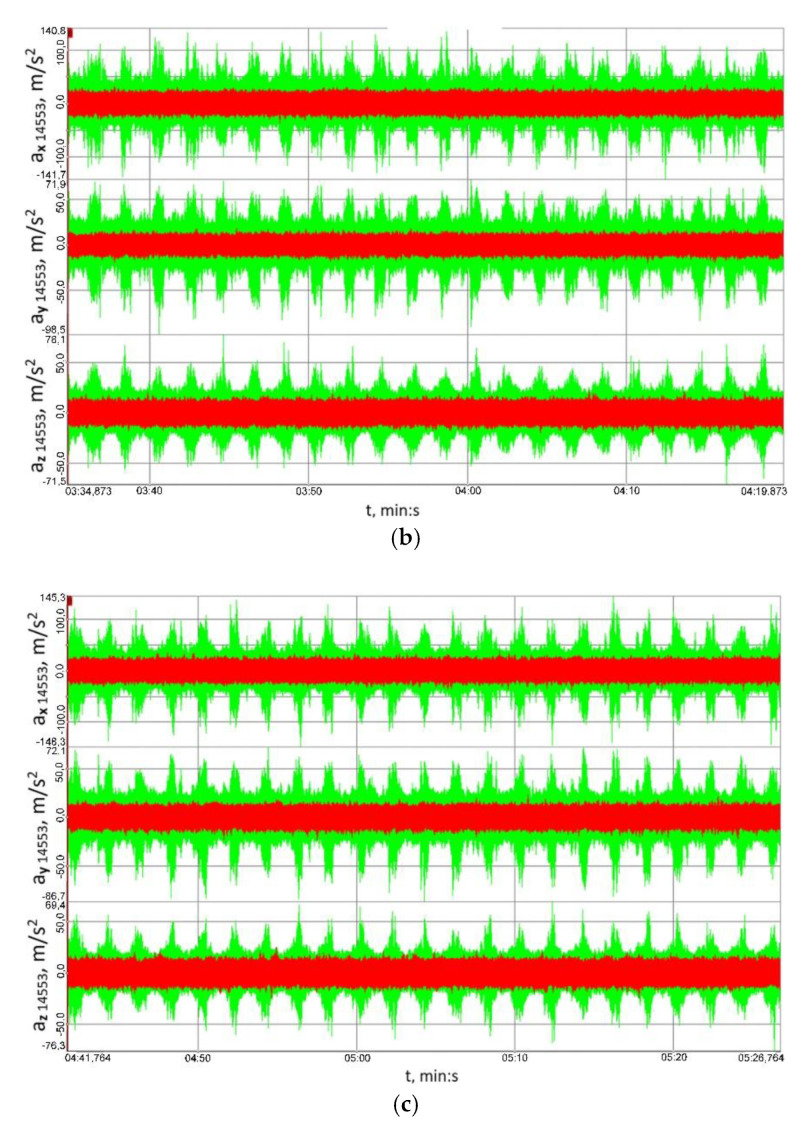
Time histories of the vibration accelerations recorded in three mutually perpendicular measurement directions using transducer No. 14553 (Figure 7) attached to the housing of the input shaft bearing in the multi-stage reduction gear: (**a**) 41.8 ± 27.9 Nm, (**b**) 79.2 ± 55 Nm, (**c**) 114.8 ± 75.9 Nm; red colour marks the results obtained for the flexible operation mode of the clutch; green colour corresponds to the locked operation mode.

Figure 12 shows the time histories of the vibration accelerations recorded in three mutually perpendicular measurement directions using transducer No. 14519 (Figure 7) attached to the housing of the output shaft bearing in the multi-stage reduction gear. Figure 13, on the other hand, shows the time histories of the vibration accelerations recorded in three mutually perpendicular measurement directions using transducer No. 14519 (Figure 7) attached to the housing of the output shaft bearing in the multi-stage reduction gear.

Having analysed the results provided in Figure 12 and Figure 13, one can clearly conclude that, for the housing of the bearing of both the input and output shaft in the multi-stage reduction gear, and for all the three loads and all the three measurement directions analysed, the peak-to-peak values of vibration accelerations obtained for the clutch in the locked operation mode were significantly lower than those obtained when the clutch was functioning in the torsionally locked operation mode. Based on these results, it can be further concluded that loads in the form of a time-varying braking torque are converted into torsional vibrations and, consequently, the linear vibrations observed on the bearing housings transferred from the meshing zone via the shafts to the bearings and their housings are lower, which is definitely a beneficial effect. 

**Figure 14:**
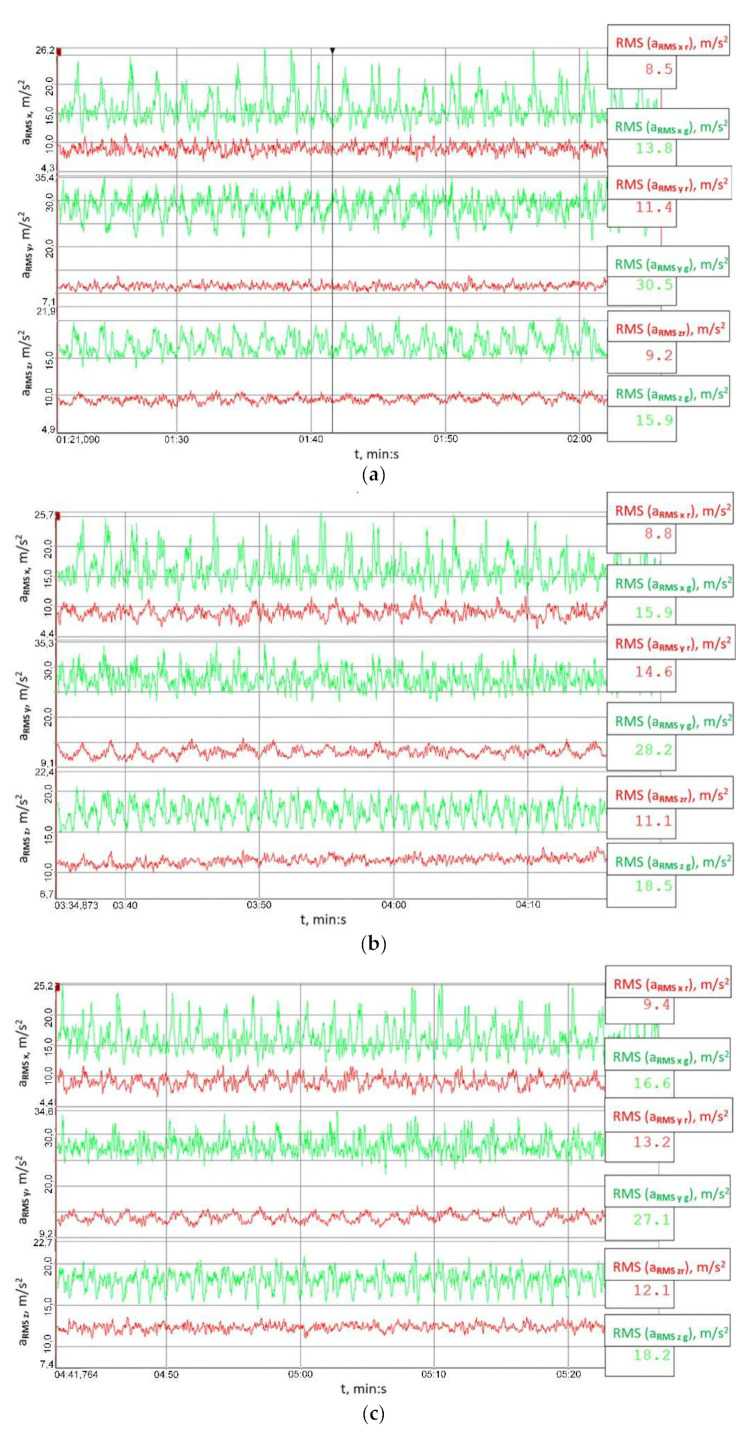
Behaviour of the RMS values of the signals recorded at the output shaft housing in the multi-stage reduction gear (point 14519) established within a time window moved along the time axis: (**a**) 41.8 ± 27.9 Nm, (**b**) 79.2 ± 55 Nm, (**c**) 114.8 ± 75.9 Nm; red colour marks the results obtained for the flexible operation mode of the clutch; green colour corresponds to the locked operation mode.

**Figure 15:**
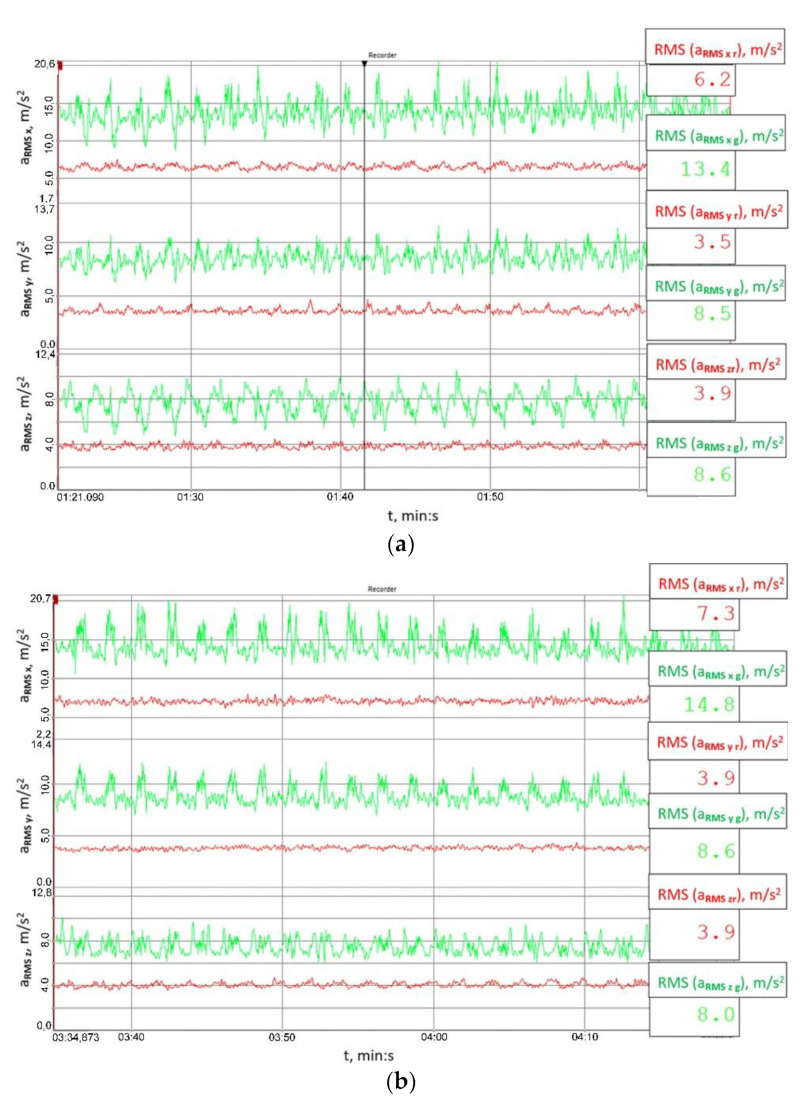

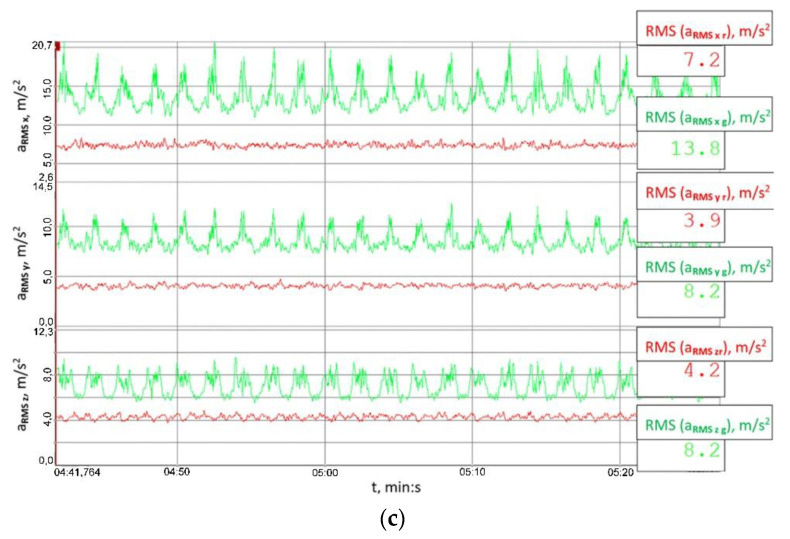
Behaviour of the RMS values of the signals recorded at the input shaft housing in the multi-stage reduction gear (point 14553) established within a time window moved along the time axis: (**a**) 41.8 ± 27.9 Nm, (**b**) 79.2 ± 55 Nm, (**c**) 114.8 ± 75.9 Nm; red colour marks the results obtained for the flexible operation mode of the clutch; green colour corresponds to the locked operation mode.

The signal’s root mean square (RMS) value is a measure of the energy of the signal, which is why the RMS values of the analysed signals were calculated, yet not straightforwardly by considering the entire behaviour of the vibration accelerations recorded, but they were established within a time window moved along the time axis. The length of the time window was 0.1 s, and the results obtained for the signals measured at the housings of the output and input shaft bearings in the reduction gear are shown in Figure 14 and Figure 15, respectively.

Figure 14 and Figure 15 explicitly imply that the RMS values of vibration accelerations calculated within the set time window were significantly lower than those obtained when the clutch was torsionally locked. This proves the high utility value of the clutch proposed for mitigating the effects of external loads on the load and vibrations of the gear.

A single toothed gear is a heavily nonlinear system, which is evidenced by the models of toothed gears discussed in the literature on the subject (as well as by the model developed by one of the co-authors of this paper [74]). Even in a one-stage toothed gear, nonlinear phenomena arise from the nonlinear rigidity of the meshing, gear play, vibration damping in the meshing, etc. The subject of the study in question is a three-stage toothed gear.

One can identify various parameters in the vibration signal generated by a one-stage toothed gear, including the following:

meshing frequency,
pinion and gear frequency,
frequencies characteristic of the operation of rolling bearings, including the following:

- BPFO (Ball Pass Frequency Outer) or outer race failing frequency;
- BPFI (Ball Pass Frequency Inner) or inner race failing frequency;
- BSF (Ball Spin Frequency) or rolling element failing frequency;
- FTF (Fundamental Train Frequency) or cage failing frequency.

**Figure 16:**
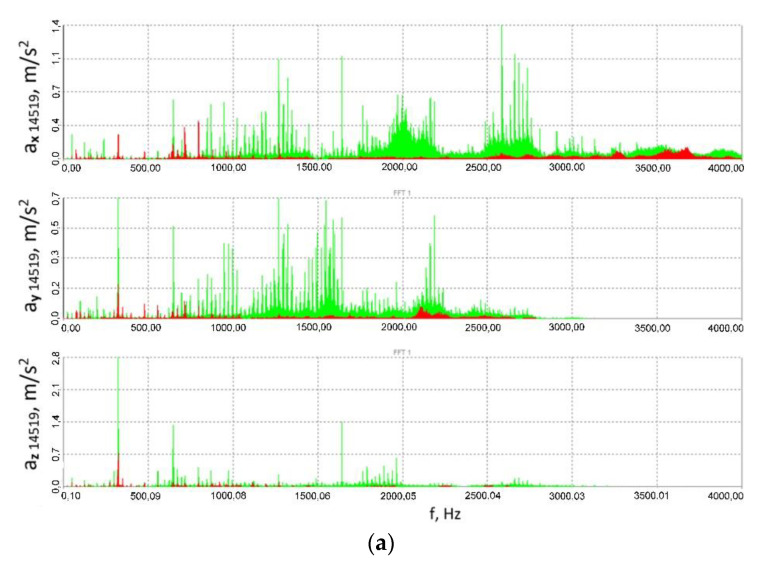

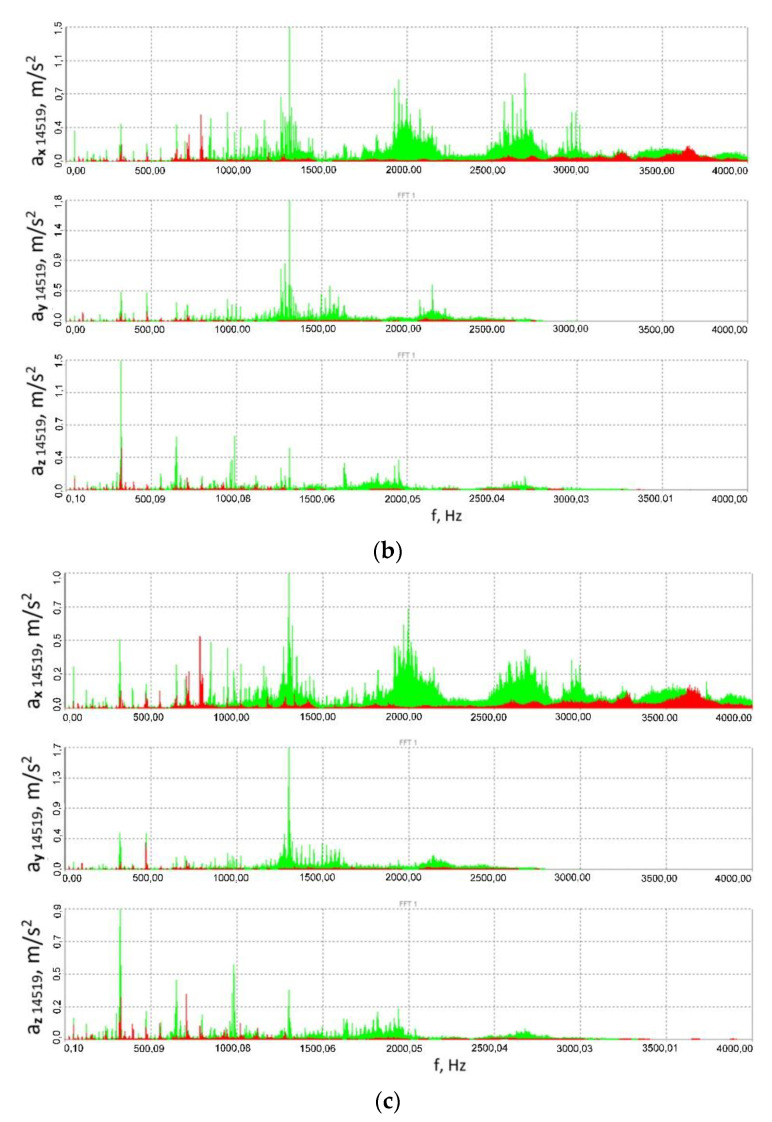
Spectra (FFT) of the vibration acceleration signals measured at the output shaft housing in the multi-stage reduction gear (point 14519): (**a**) 41.8 ± 27.9 Nm, (**b**) 79.2 ± 55 Nm, (**c**) 114.8 ± 75.9 Nm; red colour corresponds to the flexible clutch operation mode, green—locked clutch operation mode.

**Figure 17:**
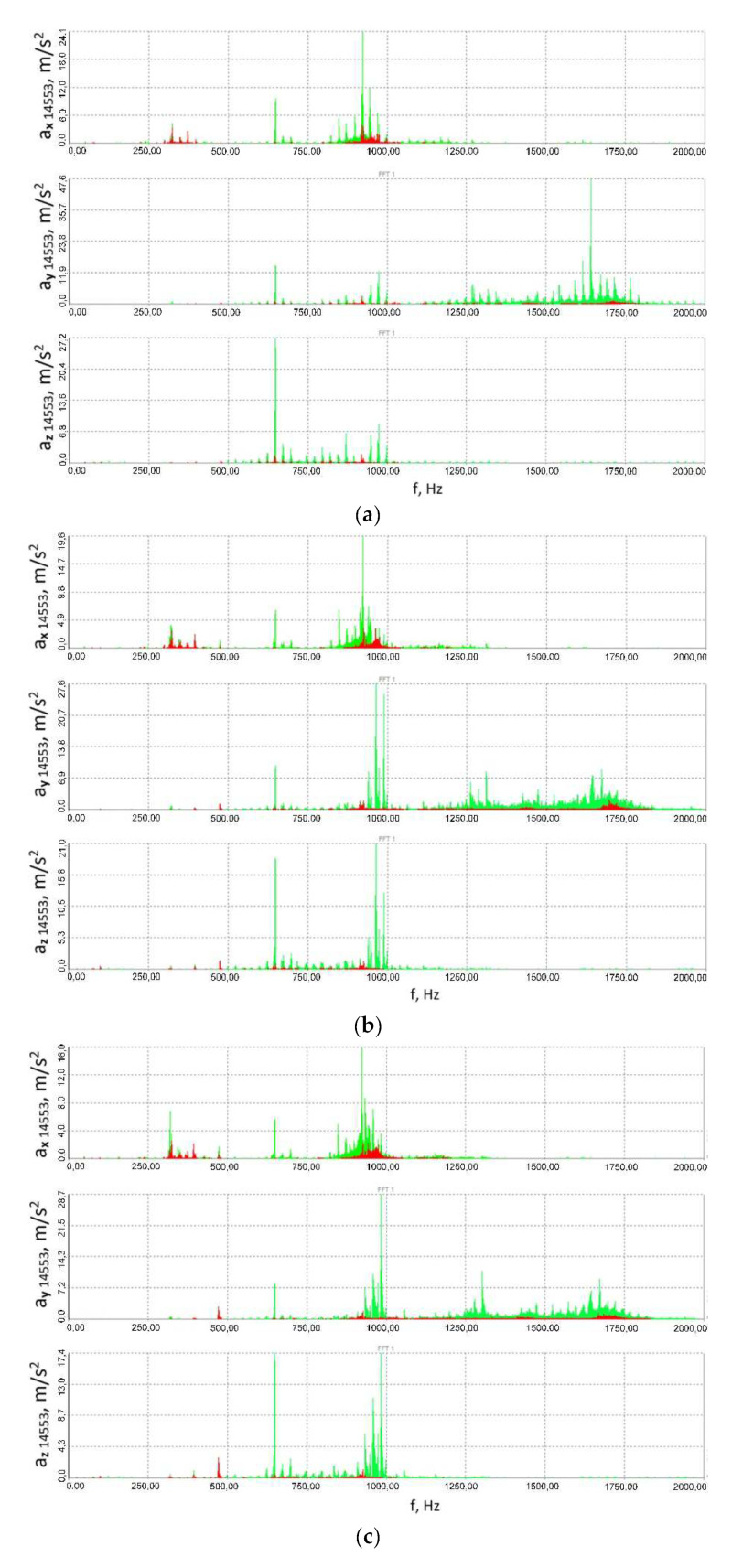
Spectra (FFT) of the vibration acceleration signals measured at the input shaft housing in the multi-stage reduction gear (point 14553): (**a**) 41.8 ± 27.9 Nm, (**b**) 79.2 ± 55 Nm, (**c**) 114.8 ± 75.9 Nm; red colour corresponds to the flexible clutch operation mode, green—locked clutch operation mode.

Furthermore, one may also deal with successive harmonics of these frequencies, and in the case of the three-stage gear subject to the studies, their number was several times greater. Additionally, what may be observed is mutual increase in the amplitude of vibrations originating at a given gear stage, characterised by a given fundamental frequency or its harmony with vibrations of identical or similar fundamental frequency (or its harmony), arising from the same or another phenomenon and another stage of the three-stage gear in question. All these input functions excite vibrations of the gear housing, which is sensitive to excitation with different frequencies. The same root mean square value of the signal or the same maximum peak-to-peak value may be caused, for instance, by vibrations of a given frequency and a high amplitude or by several signal components of different frequencies and lower amplitudes. The latter of these cases corresponds to the aforementioned subject of the studies more closely, where, for instance, one is processing a signal comprising several components of different frequencies and different amplitudes on account of a change in the system’s parameters; either the signal’s RMS value or the signal’s maximum peak-to-peak values evident in time histories may decline, but judging by the time histories one cannot be completely sure that the amplitude of each signal component has been reduced since, for example, the foregoing may be due to a significant decrease in the values of amplitudes of two signal components originally having nearly the highest amplitude; or where the value of the signal component originally having the highest amplitude has increased even more, instead of dropping, which no longer is a phenomenon that can be considered beneficial from a technical point of view and considering the effects on a physical object. Not until the FFT analysis is performed can such a doubt be dispelled, which is why spectra of the signals of accelerations of the vibrations measured at different points and in varying directions have been provided in Figure 16 and Figure 17. These spectra prove that the use of the innovative flexible clutch in question did not cause the amplitude dominant within the frequency spectrum to increase for the associated frequency in the case of the locked clutch operation, but on the contrary—it actually caused the amplitude to decline at least thrice for this frequency (depending on the measurement point and direction). 

The spectra provided in the paper prove that for the associated component frequency of the signal measured for the fixed clutch operation, the amplitude dominant within the frequency spectrum did not increase after the clutch was set to operate in the flexible mode, e.g., at the expense of decreasing the amplitudes of other frequencies, while instead it actually declined at least thrice for this frequency depending on the measurement point and direction.

Additionally, considering such a nonlinear object as a power transmission system with a toothed gear where specific modifications have been introduced and despite the fact that the overall level of vibrations has been reduced, i.e., the RMS value of the signal and the peak-to-peak values observable within the relevant time histories have been decreased, it would be advisable to use the FFT to establish whether or not the vibrations to be generated are characterised by a higher (or comparable) amplitude, yet a different frequency than that which was dominant within the spectrum and which had occurred in the test object before it was modified. Furthermore, having analysed the spectra of the accelerations of the vibrations measured at different points and in varying directions (Figure 16 and Figure 17) in the test object before and after the innovative clutch is introduced, one can observe that the use of the flexible clutch in question did not lead to reducing all the amplitudes dominant in the spectra by a fixed amplitude-reducing factor, common to all the frequencies, for any of the signals, which the authors consider to be a proof that the system is non-linear.

## 4. Conclusions

1. Using the methodology proposed by the authors of this article for testing of power transmission systems featuring the innovative and patented highly torsionally flexible metal clutch in question—a methodology which takes into account the sensing systems based on diverse phenomena: piezoelectric, magnetic, the Doppler effect, etc., as well as the amplitude and frequency analyses discussed in the paper, it can be concluded that, since the time-varying forces emerging between the mating gear teeth (including those induced by time-varying external loads) are transmitted through the shafts and bearings to the gear housing, the reduction of the vibration acceleration values of the bearing housings of both the input and output shaft in the gear owing to the use of the innovative flexible clutch in the power transmission system implies that the dynamic forces affecting the gear meshing as well as the shaft bearing have decreased, which is particularly desirable with respect to the service life of the power transmission system.
2. With reference to the complex power transmission system and based on the established instantaneous changes in the velocity of the torsional vibrations of the shaft between the clutch and the multi-stage reduction gear, illustrated in Figure 11 and recorded using a rotational laser vibrometer relatively seldom used for testing purposes, the studies addressed in this article made it possible to establish the relationships between the processes at work, i.e., the decline of the linear vibrations of the gear housing (which is undoubtedly positive in power transmission systems) at the expense of increasing torsional vibrations of the innovative clutch, the latter not to be considered unfavourable to users in the case analysed.
3. Having analysed the vibration accelerations signals observed, one can clearly conclude that, for the housing of the bearing of both the input and output shaft in the multi-stage reduction gear, and for all the three loads and all the three measurement directions analysed, the peak-to-peak values of vibration accelerations obtained for the clutch in the flexible operation mode were significantly lower than those obtained when the clutch was torsionally locked in operation. The fact that the results discussed in the paper could be obtained precisely for all three loads, two measurement points, at the input and output of the multi-stage toothed gear, and three measurement directions prior to the actual studies and analyses was actually neither obvious nor certain, but was ultimately proved, which surely is a valuable outcome.
4. The signals of the RMS values of the vibration accelerations calculated within a time window explicitly imply that, for the housing of the bearing of both the input and output shaft in the multi-stage reduction gear, and for all the three loads and all the three measurement directions analysed, the root mean square values of vibration accelerations obtained for the clutch in the flexible operation mode were significantly lower than those obtained when the clutch was torsionally locked; the foregoing proves the clutch proposed as the means to reduce the effect of external loads on the load and vibrations in the toothed gear to be highly useful.
5. Based on the results of the frequency analysis of the vibration acceleration signals for both the gear output shaft bearing housing and for that of the gear input shaft, it was established that:
  - Within the frequency bands where the vibration acceleration amplitudes showed the highest values, the vibration acceleration amplitudes were reduced multiple (at least three) times where the clutch was locked in operation as a result of the application of the patented innovative flexible clutch, this being a very beneficial effect;
  - The fact that the innovative and patented highly flexible clutch was introduced into the power transmission system did not cause that within the spectrum of the signals any amplitude emerged with a value higher than that of the amplitude dominant in the case of the locked operation of the clutch, yet associated with a frequency different than the frequency corresponding to the amplitude dominant in the case of the locked operation of the clutch.

## Figures and Tables

**Table 1:** Summary of test variants.

Mean Load Torque ± Amplitude of Load Torque Changes for Flexible Clutch Operation Mode [Nm]	Rotational Speed [rpm]	Clutch Operation Mode
41.8 ± 27.9	1480	flexible (red colour)
		locked (green colour)
79.2 ± 55		flexible (red colour)
		locked (green colour)
114.8 ± 75.9		flexible (red colour)
		locked (green colour)

**Table 2:** Specification of the Polytec RLV-5500 rotational laser vibrometer.

Rotations per Minute								
RLV-500 Sensor Head	7.5 mm beam separation				24 mm beam separation			
Measurement range	–8000 RPM–+20,000 RPM				–2500 RPM–+6500 RPM			
Analogue output	–4 V–10 V				–2.5 V–+6.5 V			
Calibration error ^1^	<0.6% of RPM reading ±2 RPM				<0.3% of RPM reading ±2 RPM			
**Angular Velocity (Δω)**								
RLV-500 Sensor	Head 7.5 mm beam separation				24 mm beam separation			
Peak analogue output (V_peak_)	10	100	1000	12,000	10	100	1000	6000
Measurement ranges (°/s/V)	±10	±10	±10	+10/−4	±10	±10	±10	+6.5/−2.5
Frequency range (kHz)	0.001–10			0–10	0.001–10			0–10
Measurement error <1% (at f = 1 kHz)								

^1^ Valid at nominal stand-off distance ± 50 mm.

## Data Availability

Not applicable.
